# Predictive value of the combined DTI-ALPS index and serum creatinine levels in mild cognitive impairment in Parkinson’s disease

**DOI:** 10.3389/fneur.2025.1628697

**Published:** 2025-08-13

**Authors:** Yuanhao Gao, Yuxin Li, Niu Ji, Pin Meng, Qing Hu, Yumei Chen, Xinying Guan, Bingchao Xu

**Affiliations:** ^1^Department of Neurology, Lianyungang Clinical College of Nanjing Medical University/The First People’s Hospital of Lianyungang, Lianyungang, China; ^2^Department of Neurology, The First People's Hospital of Lianyungang, Lianyungang, China; ^3^Department of Neurology, The Affiliated Lianyungang Hospital of Xuzhou Medical University/The First People’s Hospital of Lianyungang, Lianyungang, China

**Keywords:** Parkinson disease, mild cognitive impairment, DTI-ALPS index, serum creatinine, nomogram, prediction model

## Abstract

**Objective:**

To identify independent risk factors for Parkinson disease mild cognitive impairment (PD-MCI) and develop a prediction model integrating clinical indicators, blood biomarker, and neuroimaging data, aiding in detection and intervention.

**Methods:**

A retrospective study was conducted with 150 PD patients. The PD-MCI group (*n* = 64) and PD with normal cognition (PD-NC, *n* = 86) were identified using the Montreal Cognitive Assessment scale. Data on demographics, motor symptoms, cognitive function, quality of life, blood markers, and diffusion tensor imaging along perivascular spaces (DTI-ALPS) were collected. Univariate analysis identified significant variables, and multivariate logistic regression identified independent risk factors. A nomogram prediction model was developed using R software. Model performance was evaluated using Receiver Operating Characteristic (ROC) curves, bootstrap resampling calibration curves, and decision curve analysis (DCA).

**Results:**

Significant differences between the groups were found in levodopa equivalent daily dose (LEDD), PD Quality of Life Questionnaire, creatinine, cystatin C, and ALPS index. Multivariate regression identified higher LEDD (OR = 1.01, 95%CI 1.00–1.03, *p* = 0.005) and creatinine levels (OR = 1.34, 95%CI 1.10–1.66, *p* = 0.005) as independent risk factors. The nomogram model demonstrated strong discriminatory ability (AUC = 0.864, 95%CI 0.807–0.922) and good calibration. DCA showed a significant net benefit within clinical threshold ranges.

**Conclusion:**

This study developed a PD-MCI prediction model incorporating DTI-ALPS and clinical blood biomarkers. It confirmed that LEDD and creatinine levels are independent risk factors, with high clinical value for early screening and individualized treatment.

## 1 Introduction

Parkinson’s disease (PD), the second most common neurodegenerative disease after Alzheimer’s disease (AD), is characterized by motor symptoms accompanied by a wide range of non-motor symptoms (1). Non-motor symptoms are common throughout the disease, including anxiety, depression, cognitive impairment (CI), and autonomic dysfunction, which not only severely impact patients’ mental health and quality of life, but also significantly increase the complexity of clinical diagnosis and treatment (2). There are currently more than 10 million people with PD worldwide, and most patients experience varying degrees of CI as the disease progresses (3). Studies have shown that CI in PD patients has a complex pathological mechanism. In addition to Lewy bodies formed by abnormal aggregation of *α*-synuclein (α-syn), AD-associated biomarkers such as β-amyloid (Aβ) and tau proteins play an important role in the process of PD-induced CI (4, 5). These pathological changes ultimately lead to progressive cognitive decline by inducing neuronal damage, disrupting synaptic function and compromising the integrity of neural networks. CI typically progresses through three stages: subjective cognitive decline, mild cognitive impairment (MCI), and Parkinson’s disease dementia (PDD). Of these, MCI is considered a prodromal state in the development of dementia, representing the transition between normal aging and marked cognitive dysfunction. Epidemiological studies have shown that approximately 80% of people with Parkinson’s disease will develop dementia within 20 years of diagnosis (6). Similarly, studies have shown that approximately 50% of PD patients with normal cognition at baseline will develop cognitive impairment within 6 years, and all new cases of MCI will progress to dementia within 5 years (7).

Presently, the diagnosis of PD-MCI is undergoing a shift toward multimodal assessment, integrating a range of methodologies including single photon emission computed tomography imaging of dopamine transporters, the detection of α-syn and AD markers in cerebrospinal fluid, as well as neurophysiological and genetic analysis (8). Nevertheless, there are still many challenges in reality: the prevalence of advanced magnetic resonance imaging (MRI) sequence examinations is relatively low, the risk of invasive operation in cerebrospinal fluid collection, and the high cost of the examination also limit its wide application. Consequently, subjective assessment tools such as Montreal Cognitive Assessment (MoCA) scale remain the prevailing standard in clinical practice. In this context, there is an urgent need to construct a multidimensional prediction model based on routine examination tools to achieve early identification of PD-MCI, facilitate intervention and improve prognosis. The model constructed in previous studies (9), combining neuron-specific enolase and nigrostriatal hyperchromicity, demonstrated high accuracy in predicting CI associated with PD (AUC = 0.823, 95% CI 0.781–0.864, *p* < 0.001). This study also suggests that integrating clinical indicators with neuroimaging biomarkers can help improve the identification of CI in patients with PD.

It is worthy of note that recent interdisciplinary studies have found evidence to suggest a potential association between the pathological processes of neurodegenerative diseases and renal function abnormalities. In particular, the abnormal phosphorylation of pathological α-syn in renal parenchymal cells may constitute a key link in the propagation of the “kidney-brain axis” pathology (10). Multiple cohort studies have confirmed that inflammatory markers such as plasma C-reactive protein (CRP) (11) and the specific proteins of the central nervous system (S100) protein family (12), as well as renal function parameters including creatinine (Cr) (13), cystatin C (Cys C) (14), and estimated glomerular filtration rate (eGFR), are associated with PD. The glymphatic system serves as a crucial pathway for interstitial fluid circulation and metabolic waste clearance in the central nervous system. Traditionally, studies have employed linear gadolinium-based contrast agents administered intrathecally or intravenously, followed by time-resolved MRI to track their distribution and evaluate glymphatic drainage in the brain (15). However, these methods are invasive and carry potential risks. In recent years, a promising non-invasive technique known as diffusion tensor image analysis along the perivascular space (DTI-ALPS) has been developed. First introduced by Taoka et al. (16), this approach utilizes MRI-DTI sequences to measure the anisotropic diffusion of water molecules in the brain’s white matter, thereby enabling indirect functional assessment of the cerebral glymphatic system. The ALPS index is calculated by placing regions of interest (ROI) at the intersections where periventricular veins cross perpendicularly with white matter fibers, including projection and association fibers. Diffusivity values are extracted along various directions, and the ratio of diffusivity along the perivascular direction to that in the perpendicular direction is computed. This index reflects the efficiency of fluid movement along perivascular spaces within the brain and can be used as an indirect measure of the functional status of the brain’s glymphatic system (17).

Many clinical trials are currently underway to explore potential disease-modifying therapeutic strategies for PD, in the hope of halting or significantly slowing the neurodegenerative process (18). However, most studies on PD-MCI focus on the correlational analysis of influencing factors, and the practical applications for disease prevention and early detection are still limited. Additionally, there is a lack of studies assessing the risk of PD-MCI through the construction of clinical prediction models. In conclusion, this study integrates routine multidimensional data, including clinical assessments, hematological tests, and imaging indices, to construct a clinical model for predicting PD-MCI risk. This model aids in early identification of high-risk individuals and provides a foundation for early screening and delayed cognitive decline.

## 2 Materials and methods

### 2.1 Study type

It was a single-center retrospective study.

### 2.2 Research subjects

This study was approved by the Ethical Committee of the First People’s Hospital of Lianyungang (approval number: KY-20220812002-01), and all participants provided written informed consent prior to enrollment. Involving 178 PD patients who were admitted to the Department of Neurology at the First People’s Hospital of Lianyungang between January 2023 and June 2024. All patient diagnoses adhered to the revised Parkinson’s Disease Society Brain Bank criteria and were confirmed by at least two consultants using a double-blind method. Cognitive function was assessed using the standard MoCA scale, following Movement Disorder Society guidelines. The Level II comprehensive neurological psychological assessment protocol was employed to evaluate PD-related cognitive characteristics, ensuring study rigor and result comparability (19).

Inclusion criteria for this study required subjects to comply with cognitive-behavioral treatment, to complete neuropsychological assessment and multimodal imaging, and to have disease duration of ≥1 year from initial diagnosis. Exclusion criteria included: (1) missing key data; (2) secondary Parkinson’s syndrome or superimposed Parkinson’s syndrome; (3) intracranial space-occupying lesions; (4) psychoneurological disorders due to severe cerebrovascular disease, traumatic brain injury and other diagnoses; and (5) Hoehn-Yahr stage (H-Y stage) > 3 or the presence of motor/consciousness deficits.

After a standardized screening process, 7 cases were excluded due to loss to follow-up; 13 chose to withdraw because of their own movement disorders, which prevented them from cooperating with MRI examinations and clinical evaluations; and 6 cases were excluded due to motion artifacts or substandard image quality during the imaging process. After the above screening, a total of 150 PD patients successfully completed the entire assessment process and were included in the subsequent research analysis. Given the high specificity of MoCA in identifying PD-MCI, a score of 26 was used as the cut-off in this study: patients with a score greater than 26 were included in the normal cognition group (NC group), and patients with a score between 16 and 26 were classified as the PD-MCI group (20, 21).

### 2.3 Collection of clinical data

Baseline demographic data including age, sex, education time and disease duration were collected, and body mass index (BMI) (weight [kg]/height [m]^2^) was calculated using standardized anthropometric methods as an indicator of metabolic status. Doses of antiparkinsonian medication were converted to levodopa equivalent daily doses (LEDD) according to the formula recommended by the International Movement Disorders Society (22). The neuropsychiatric assessment system included the Hamilton Anxiety Scale (HAMA) and the Hamilton Depression Scale (HAMD) to quantify anxiety and depressive symptoms, respectively, and the Pittsburgh Sleep Quality Index (PSQI) to assess sleep rhythm disturbances. PD status was assessed by a systematic multidimensional analysis using the Unified Parkinson’s Disease Rating Scale (UPDRS), which includes four subscales to quantify non-motor symptoms (Section I), activities of daily living (Section II), motor signs (Section III), and motor complications (Section IV). Meanwhile, the modified (H-Y stage) was used to objectively assess symptom severity and staged disease progression. In addition, the PD Quality of Life Questionnaire (PDQ-39) and the Scale for the Assessment of Autonomic Function (SCOPA-AUT) were used together to comprehensively assess patients’ health-related quality of life and autonomic dysfunction characteristics, and a comprehensive scoring system covering symptom dimensions, functional status and quality of life was constructed.

### 2.4 Peripheral examination

Peripheral blood test indices including total neutrophil count, C-reactive protein (CRP), and S100-*β* were recorded during the patients’ hospitalization. Renal function-related indices included Cr and Cys C. All subjects had their peripheral blood specimens collected through elbow vein in the early morning fasting state, and the serum was separated by centrifugation at 3500 rpm for 6 min, and then immediately placed at −20°C for freezing and storage. The serum samples were equilibrated and rewarmed at room temperature and vortexed thoroughly before testing. Cys C was measured by latex-enhanced immunoturbidimetric assay, and Cr was measured by enzymatic assay. Both assays were performed on a fully automated biochemical analyzer, and the whole process was carried out in strict accordance with standardized operating procedures, and comprehensive quality control measures were implemented.

### 2.5 MRI image acquisition

Image data acquisition was performed by two radiologists with more than 5 years of neuroimaging experience to ensure image quality. Subjects were required to abstain from anti-Parkinsonian medication for at least 12 h prior to scanning. The MRI was performed using a 3.0 T Siemens Prisma scanner in the imaging department of the First People’s Hospital of Lianyungang. The parameters of the T1-weighted images (T1WI) scan were as follows: repetition time = 3,800 ms, echo time = 90 ms, flip angle = 70°, slice thickness = 6 mm, slice spacing = 1 mm, field of view = 210 mm, matrix size = (210 × 220), and number of scanned slices = 92. The parameters of the DTI scan were as follows: SE-EPI sequence imaging, repetition time = 9,300 ms, echo time = 69 ms, slice thickness = 1.5 mm, slice spacing = 0 mm, field of view = 256 mm × 256 mm × 100, matrix = 110 × 110, 64 diffusion-weighted scans were performed with b = 1000s/mm^2^ and another diffusion-weighted scan with b = 0 and another diffusion-weighted scan with b = 1,000 s/mm^2^ and another diffusion-weighted scan with b = 0. Another diffusion-weighted image was performed with b = 0 and number of excitations = 2. The total scan time was 8 min 35 s.

### 2.6 DTI-ALPS processing

A standardized DTI data processing procedure was used for the study. After the raw image data in DICOM format were uniformly converted to NIFTI format using the dcm2niix tool, batch processing was performed using the Diffusion Toolkit^1^: first, head motion correction was performed to remove motion artifacts, followed by extracranial tissue stripping using the Brain Tissue Extraction (BET) algorithm, and then voxel-level diffusion tensor fitting was performed using the DTIFIT model to obtain maps of anisotropy fraction (FA), mean diffusivity (MD), principal eigenvalues (λ1–λ3), and diffusion tensor component (Dxx/Dyy/Dzz) parameters. Based on the eigenvalue parameters, radial diffusion coefficient (RD) maps were calculated using the (λ2 + λ3)/2 equation. For the calculation of the DTI-ALPS index, a 5 mm diameter ROI region was manually outlined in the region of the intersection of projection and contact fibers in the bilateral cerebral hemispheres based on color-coded FA maps using the FSLeyes (version 1.14.2) tool^2^. By measuring the *x*-axis (Dxxproj) and *y*-axis (Dyyproj) diffusion coefficients in the projection fiber region and the *x*-axis (Dxxassoc) and *z*-axis (Dzzassoc) diffusion coefficients in the contact fiber region, the ALPS indices of the bilateral cerebral hemispheres were calculated, The ALPS indices of the bilateral cerebral hemispheres were calculated separately according to Taoka’s method (16), and the final average value of the bilateral hemispheres was taken as the ALPS index for the comprehensive assessment of the structural integrity of the cerebral fiber-vessel unit. ALPS index.

The calculation formula is as follows: $\text{ALPS}-\text{Index}=\frac{\text{mean}(\text{Dxxproj},\text{Dxxassoc})}{\text{mean}\;(\text{Dyyproj},\text{Dzzassoc}).}$

### 2.7 Statistical methods

SPSS 26.0 with R 4.5.0 was used for statistical analysis and model building in this study.

Normally distributed measures were expressed as mean (standard deviation) [Mean (SD)] and *t*-test was used to compare between groups; non-normally distributed data were expressed as median (interquartile range) [Median (IQR)] and Mann–Whitney *U*-test was used; and the categorical variables (Sex, H-Y stage) were described as frequency (percentage) [*n* (%)] and Chi-square test was used. Variables that were statistically significant (*p* < 0.05) in the univariate analysis were included in the multifactorial logistic regression analysis to screen for independent risk factors for PD-MCI. Based on the results of the multifactorial logistic regression analysis, a column-line graph prediction model was constructed using the rms program package in the R language to quantify the risk factor weights. Model discrimination was assessed by receiver operating characteristic (ROC) curve, and internal validation was performed by bootstrap 1,000 resampling, and model calibration and clinical net benefit were analyzed by calibration curve and decision curve, respectively. The significance level was set at *α* = 0.05 (two-tailed test).

## 3 Results

### 3.1 Baseline demographic information and clinical data

**Figure 1:**
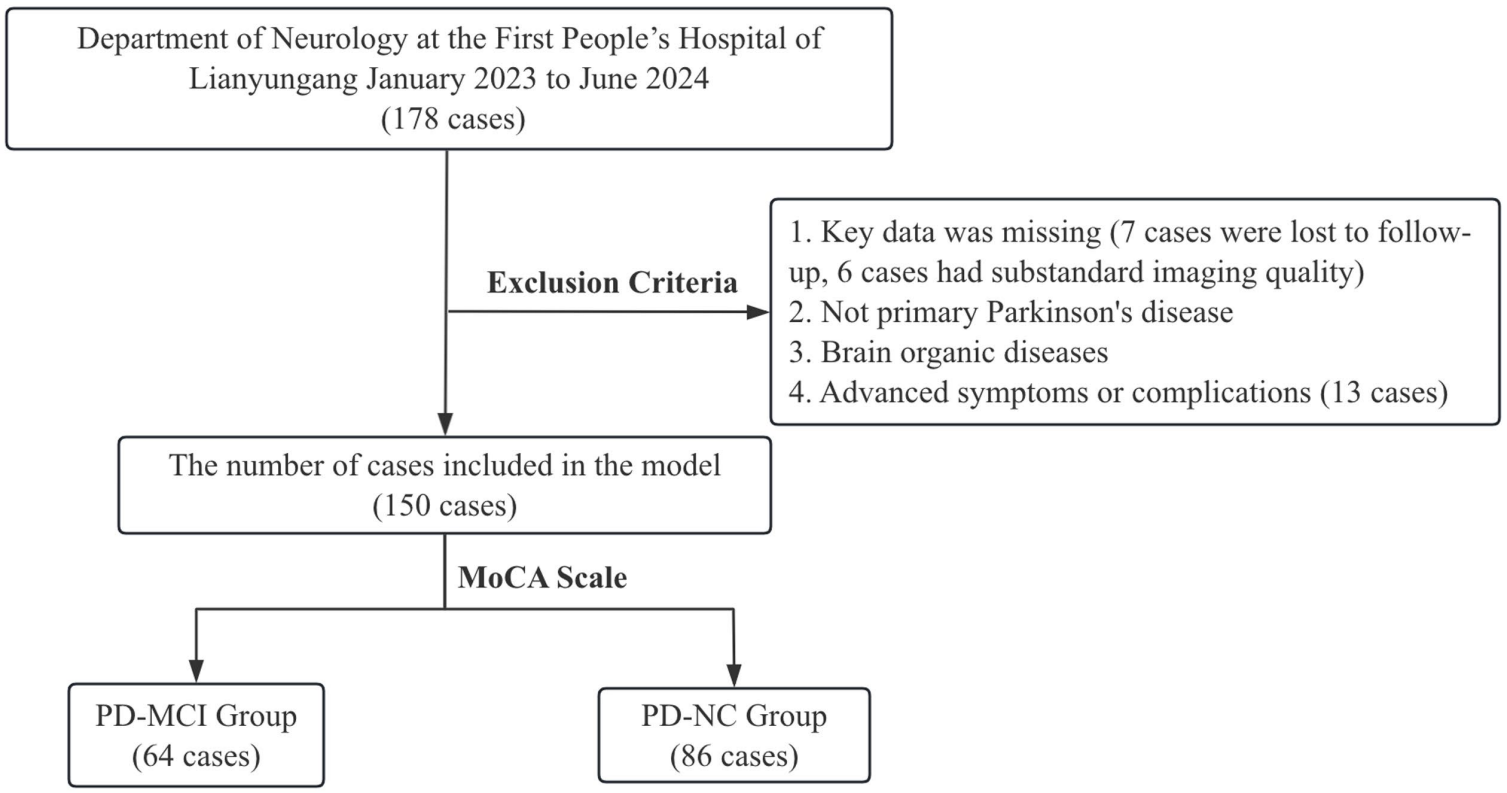
Study flowchart.

A total of 150 PD patients who met the enrolment criteria were included in this study and cognitively stratified by MoCA, including 64 patients in the PD-MCI group and 86 patients in the PD-NC group (Figure 1). The baseline characteristics of the two groups (Table 1), showed statistically significant differences between the LEDD, PDQ-39, UPDRS I, Cr, Cys C and ALPS index (*p* < 0.05).

**Table 1:** Comparison of baseline data between PD-MCI group and PD-NC group.

Variables	PD-NC (*n* = 86)	PD-MCI (*n* = 64)	*p*-value
Age (year) Mean (SD)	68.92 (4.30)	69.61 (5.05)	0.37^a^
Sex			0.78^c^
Female	41 (47.67)	32 (50.00)	
Male	45 (52.33)	32 (50.00)	
BMI (kg/m^2^) Mean (SD)	21.79 (1.91)	21.30 (2.00)	0.13^a^
Education time (year) Median (IQR)	9.00 (6.00, 9.00)	6.00 (6.00, 9.00)	0.40^b^
Disease duration (year) Median (IQR)	3.00 (2.00, 5.00)	3.00 (2.00, 4.25)	0.72^b^
LEDD (mg) Mean (SD)	399.34 (45.21)	449.95 (44.49)	**<0.01** ^a^
H-Y Stage			0.91^c^
Stage 1	15 (17.44)	12 (18.75)	
Stage 2	51 (59.30)	39 (60.94)	
Stage 3	20 (23.26)	13 (20.31)	
UPDRS I Mean (SD)	6.50 (2.67)	8.27 (2.76)	**<0.01** ^a^
UPDRS II Median (IQR)	9.00 (7.00, 10.00)	9.00 (8.00, 12.00)	0.17
UPDRS III Mean (SD)	28.10 (4.56)	29.38 (4.55)	0.09^a^
PDQ-39 Median (IQR)	35.00 (32.00, 40.00)	43.00 (38.00, 51.25)	**<0.01** ^b^
SCOPA-AUT Mean (SD)	17.45 (3.65)	17.17 (3.50)	0.64^a^
MoCA Mean (SD)	28.07 (1.03)	19.55 (2.67)	**<0.01** ^a^
HAMA Median (IQR)	9.00 (5.25, 12.75)	10.00 (6.75, 15.25)	0.20^b^
HAMD Median (IQR)	10.00 (6.25, 13.00)	9.00 (6.00, 14.00)	0.64^b^
PSQI Median (IQR)	9.00 (7.00, 11.00)	10.00 (8.00, 13.25)	0.08^b^
Neutrophil count (10^9^/L) Mean (SD)	3.80 (0.98)	3.72 (0.83)	0.62^a^
CRP (mg/L) Mean (SD)	7.22 (1.11)	7.08 (0.82)	0.38^a^
Cr (μmol/L) Mean (SD)	68.23 (2.19)	70.42 (2.06)	**<0.01** ^a^
Cys C (mg/L) Mean (SD)	0.95 (0.12)	1.06 (0.12)	**<0.01** ^a^
S100-β (ng/ml) Median (IQR)	0.10 (0.10, 0.11)	0.10 (0.10, 0.10)	0.52^b^
L-ALPS Mean (SD)	1.37 (0.12)	1.30 (0.08)	**<0.01** ^a^
R-ALPS Median (IQR)	1.33 (1.26, 1.38)	1.31 (1.22, 1.40)	0.20^b^
ALPS Index Mean (SD)	1.35 (0.10)	1.31 (0.07)	**<0.01** ^a^

Education time, Disease duration, UPDRS II, PDQ-39, HAMA, HAMD, PSQI, S100-β are presented as the median (interquartile range); Sex and H-Y Stage are presented as number (frequency). The other parameters are presented as the mean (standard deviation). ^a^Unpaired independent *t*-test; ^b^non-parametric Mann–Whitney test; ^c^*χ*^2^ test. BMI, Body Mass Index; LEDD, Levodopa Equivalent Daily Dose; H-Y Stage: Hoehn and Yahr Stage; UPDRS I/II/III, Unified Parkinson’s Disease Rating Scale part I/II/III; PDQ-39, Parkinson’s disease Quality of Life Questionnaire; SCOPA-AUT, Scale for the Assessment of Autonomic Function; MoCA, Montreal Cognitive Assessment; HAMA: Hamilton Anxiety Rating; HAMD, Hamilton Depression Rating; PSQI, Pittsburgh Sleep Quality Index; CRP, C-reactive Protein; Cr, Creatinine; Cys C: Cystatin C; S100-β: specific proteins of the central nervous system protein beta; ALPS Index: diffusion tensor image analysis along the perivascular space index. *P*-values < 0.05 were considered statistically significant.

### 3.2 Multivariate logistic regression analysis of PD-MCI patients

**Figure 2:**
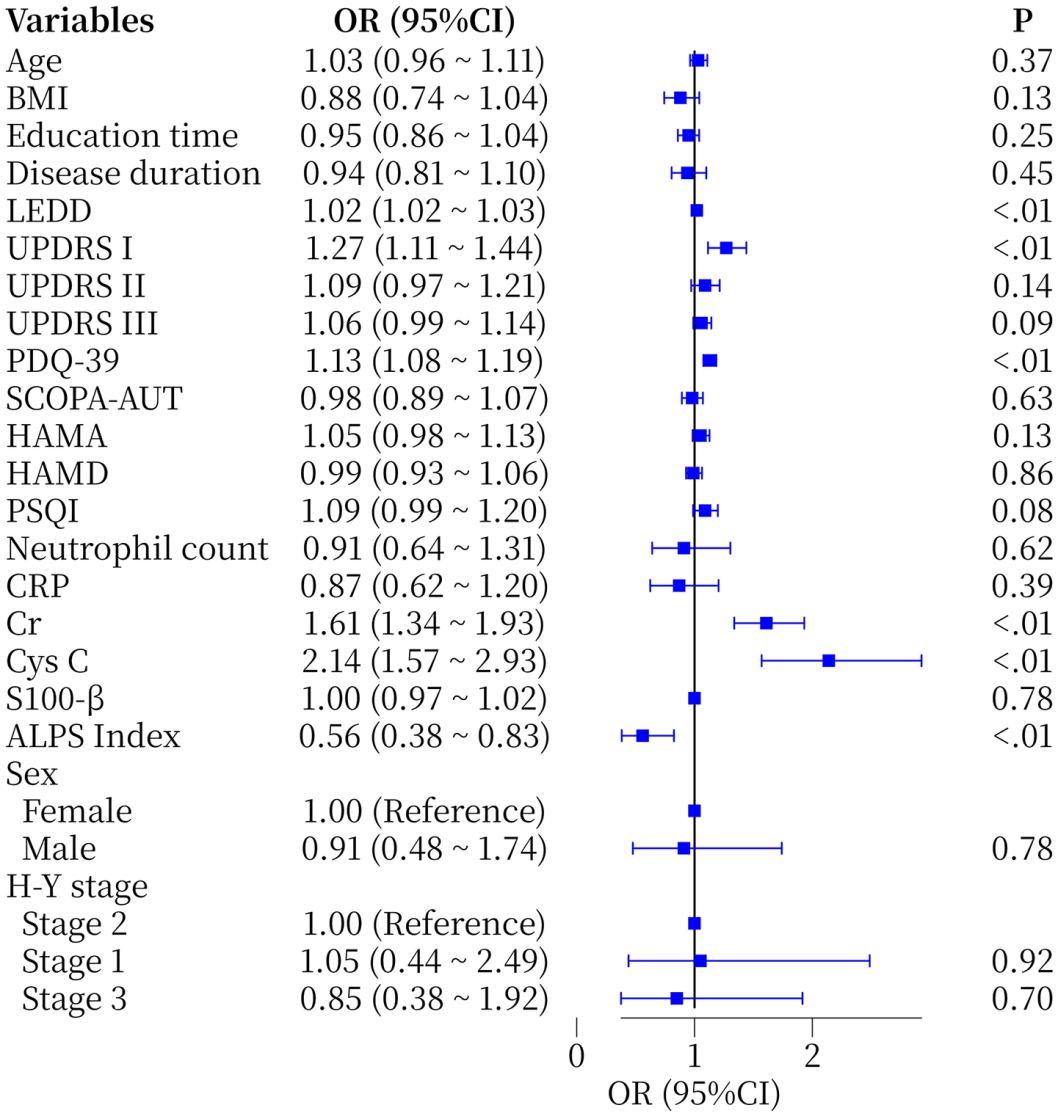
Forest plot of single-factor logical regression analysis of the occurrence of MCI in PD patients. BMI, Body Mass Index; LEDD, Levodopa Equivalent Daily Dose; H-Y Stage: Hoehn and Yahr Stage; UPDRS I/II/III, Unified Parkinson’s Disease Rating Scale part I/II/III; PDQ-39, Parkinson’s disease Quality of Life Questionnaire; SCOPA-AUT, Scale for the Assessment of Autonomic Function; HAMA: Hamilton Anxiety Rating; HAMD, Hamilton Depression Rating; PSQI, Pittsburgh Sleep Quality Index; CRP, C-reactive Protein; Cr, Creatinine; Cys C, Cystatin C; S100-β, specific proteins of the central nervous system protein beta; ALPS Index, diffusion tensor image analysis along the perivascular space index.

One-way logistic regression showed that the Cr (OR = 1.61, 95%CI 1.34–1.93, *p* < 0.01) and ALPS index (OR = 0.56, 95%CI 0.38–0.83, *p* < 0.01) were statistically significant (Figure 2). Furthermore, whether PD patients had comorbid MCI or not was used as a dependent variable (1 = yes, 0 = no) and variables with *p* < 0.05 in univariate analysis (LEDD, PDQ-39, UPDRS I, Cr, Cys C and ALPS index were included). A multivariate model was constructed using backward stepwise regression, which showed that LEDD (OR = 1.01, 95%CI 1.00–1.03, *p* = 0.005) and Cr (OR = 1.34, 95%CI 1.10–1.66, *p* = 0.005) retained their independent predictive value. Notably, although PDQ-39, Cys C and ALPS index were significant in the univariate model, they did not show independent associations in the multivariate model (Table 2), suggesting that their effects may be influenced by confounders or attenuated by the limited sample size.

**Table 2:** Multivariate logistic regression analysis of risk factors for PD-MCI.

Variables	OR	S. E.	*Z*	95%CI	*P-*value
LEDD	1.01	0.005	2.81	1.00, 1.03	**0.005**
PDQ_39	1.06	0.032	1.90	1.00, 1.13	0.058
Cr	1.34	0.104	2.81	1.10, 1.66	**0.005**
Cys C	1.36	0.197	1.55	0.93, 2.01	0.121
ALPS Index	0.68	0.253	−1.50	0.41, 1.11	0.133

OR, odds ratio; S. E., Standard Error; Z, Regression coefficient; CI, confidence interval. LEDD, Levodopa Equivalent Daily Dose; PDQ-39, Parkinson’s disease Quality of Life Questionnaire; Cr, Creatinine; Cys C, Cystatin C; ALPS Index, diffusion tensor image analysis along the perivascular space index. *P*-values < 0.05 were considered statistically significant.

### 3.3 Construction of the PD-MCI risk nomogram prediction model

**Figure 3:**
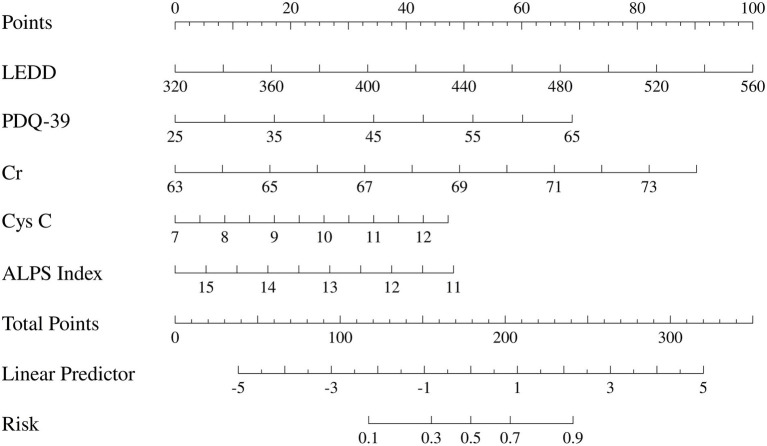
Presents the constructed nomogram to visually display the individual probabilities for the diagnosis of PD-MCI. LEDD, Levodopa Equivalent Daily Dose; PDQ-39, Parkinson’s disease Quality of Life Questionnaire; Cr, Creatinine; Cys C, Cystatin C; ALPS Index, diffusion tensor image analysis along the perivascular space index.

Based on the results of the multivariate logistic regression analysis, a nomogram model for predicting the risk of MCI in patients with PD was constructed using the R software (Figure 3).

### 3.4 Validation of the PD-MCI risk nomogram prediction model

**Figure 4:**
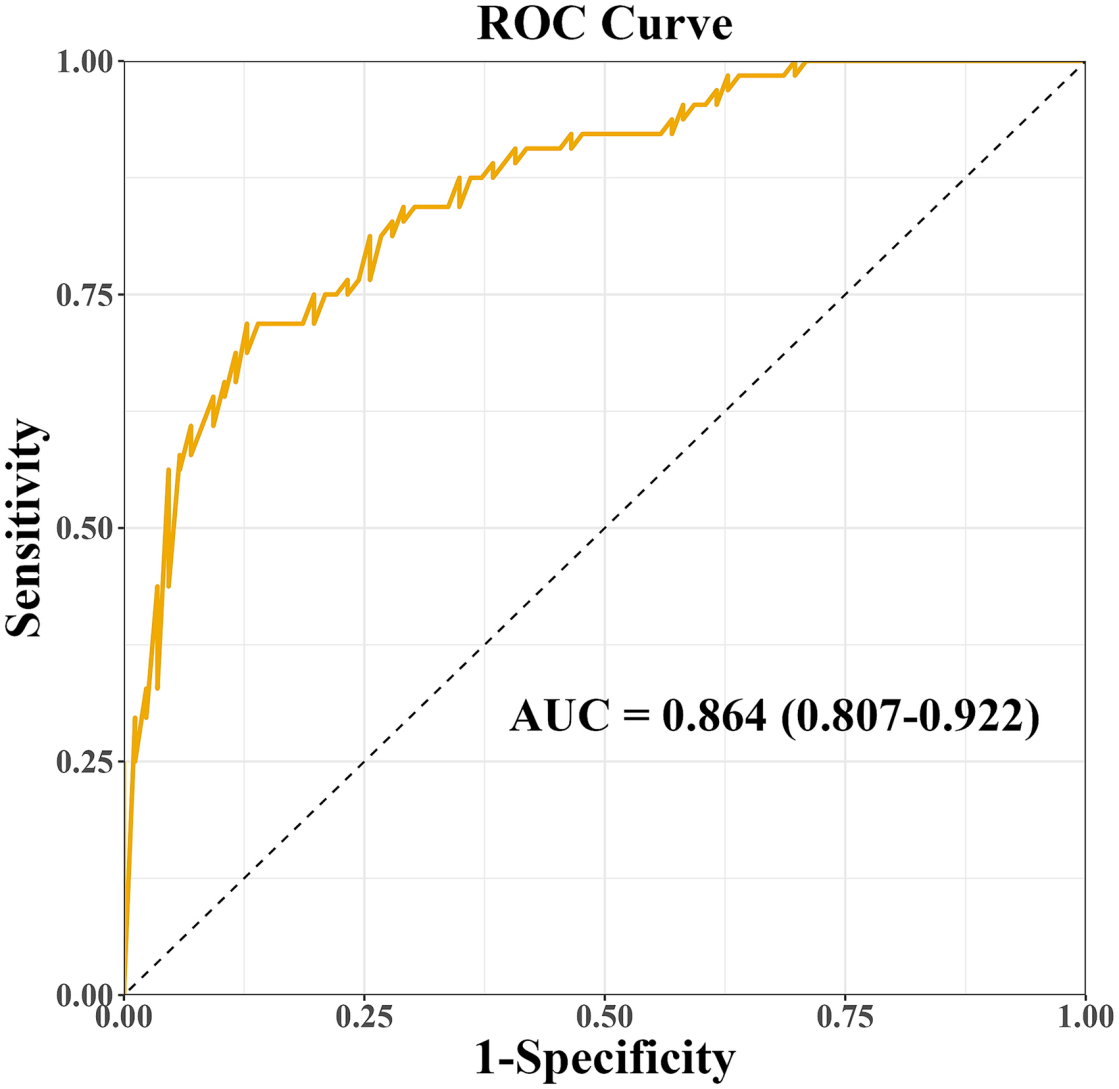
Receiver operating characteristic (ROC) curve of the nomogram model for predicting PD-MCI risk. AUC, area under the curve.

**Figure 5:**
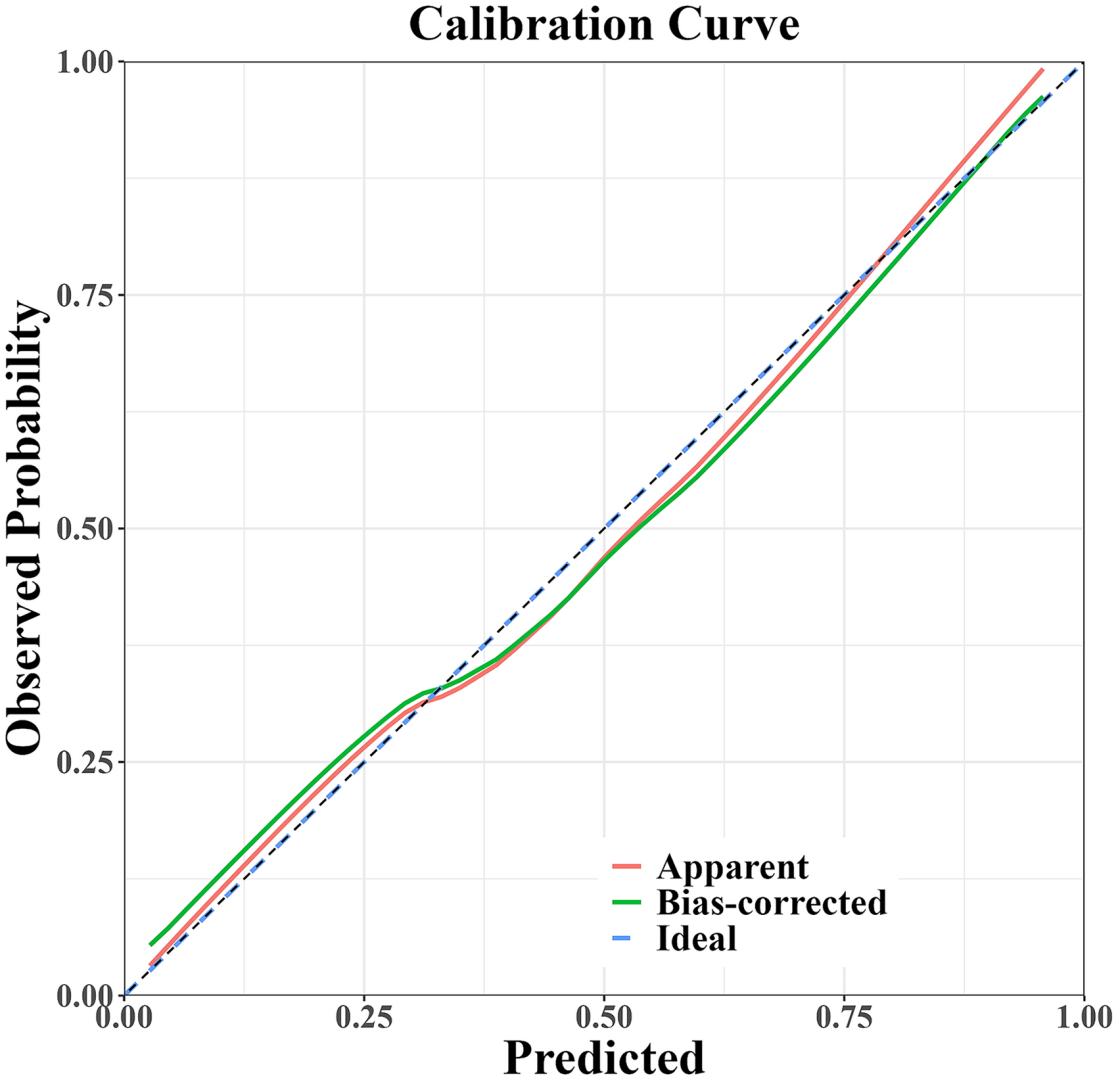
Calibration curve of the PD-MCI risk prediction model.

**Figure 6:**
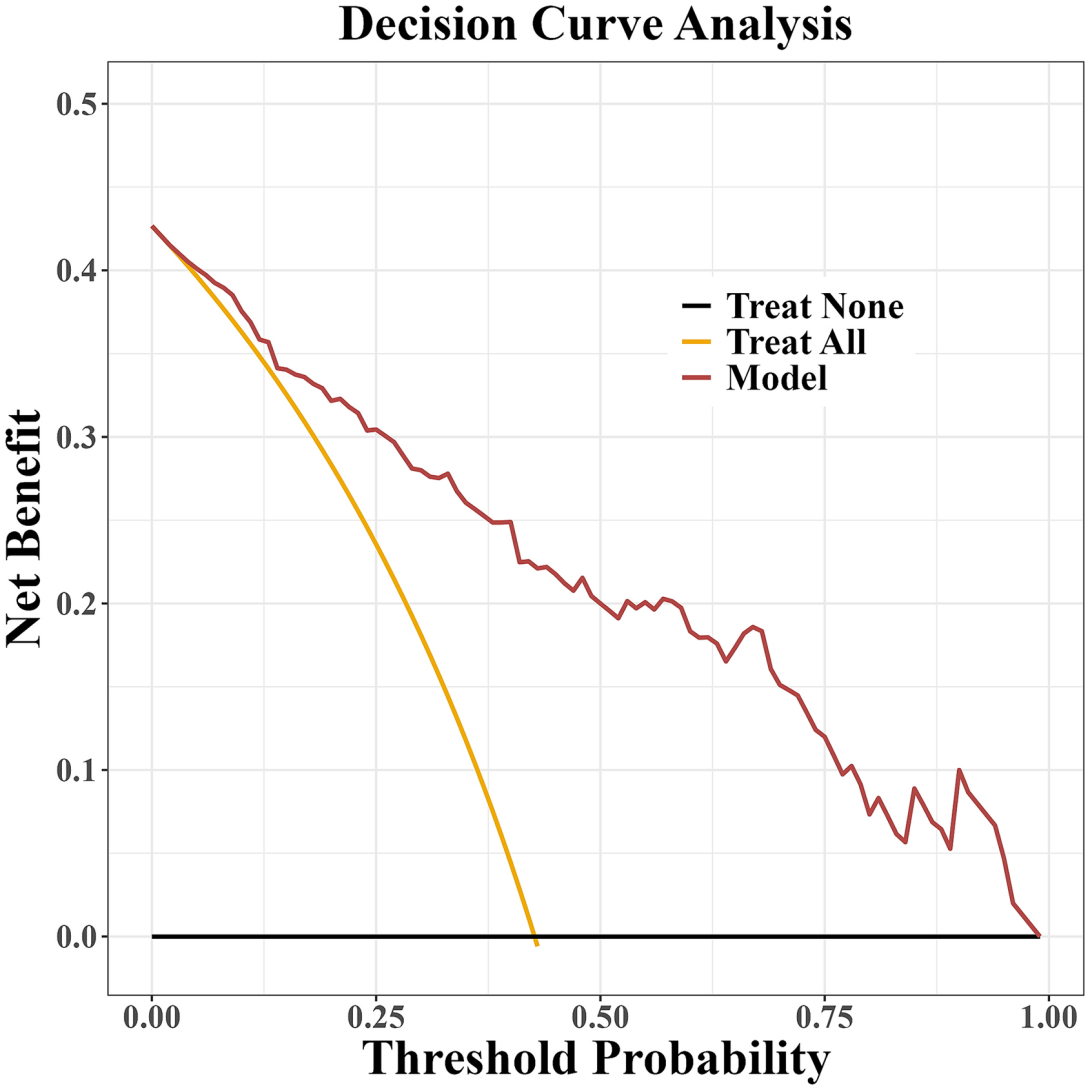
Decision curve analysis of the PD-MCI risk prediction model.

The prediction model developed in this study demonstrated strong performance in terms of discriminatory ability, calibration, and clinical applicability. ROC curve analysis revealed an AUC of 0.864 (95% CI: 0.807–0.922), indicating excellent discriminatory power for predicting the risk of PD-MCI. At the optimal cut-off, the model showed high sensitivity (0.87) and specificity (0.81), effectively identifying high-risk individuals (Figure 4). Calibration results, assessed by the Hosmer-Lemeshow test, confirmed no significant deviation between predicted probabilities and observations, with the calibration curve closely following the ideal curve, indicating good calibration accuracy (Figure 5). To evaluate robustness and prevent overfitting, we conducted 1,000 bootstrap resampling validations, showing consistency in key predictive parameters across resamples. The calibration curves from these validations mirrored those of the original model, supporting its robustness. Decision curve analysis demonstrated a net benefit over standard practice when the decision threshold was set between 0.05 and 0.75, confirming the model’s clinical utility, particularly for low and intermediate-risk stratification. However, in scenarios with higher thresholds, the relatively small high-risk subgroup sample size may introduce greater variability (Figure 6).

## 4 Discussion

The objective of this study was to curtail the reliance on high-cost specialized tests by optimizing the clinical assessment process, focusing on admission routine testing indicators, and combining brain structure and white matter fiber bundle integrity parameters obtained by multimodal MRI to assess the function of the glymphatic system based on the ALPS index. A multidimensional prediction model of PD-MCI was constructed by integrating and analyzing clinical features, blood biomarkers and neuroimaging indexes. Multifactorial logistic regression analysis demonstrated that LEDD, PDQ-39, Cr, Cys C and ALPS index were significantly associated with the occurrence of PD-MCI (*p* < 0.05). Among the identified predictors, LEDD (OR = 1.01, 95%CI 1.00–1.03) and Cr (OR = 1.34, 95%CI 1.10–1.66) were found to be independent predictors. The column-line graph model demonstrated excellent predictive efficacy, with an AUC of 0.864 (95%CI 0.807–0.922). Internal validation was conducted using the bootstrap method with 1,000 iterations, and the corrected C-index was maintained at 0.864. This suggests that the model is stable and that the risk of overfitting is controllable. The calibration curve analysis demonstrated that the predicted probabilities exhibited a high degree of consistency with the actual observations, and the decision curve analysis further confirmed that the model had the capacity to provide significant net clinical benefits within the risk probability threshold of 5–75%. It is important to note that in the high-risk prediction interval with a risk threshold greater than 0.75, the MoCA scores of the CI group in this study were not centrally distributed in the threshold interval, suggesting severe cognitive impairment. Furthermore, the limited sample size of high-risk subgroups, such as PDD, may increase the variability of the prediction estimates and thus reduce the decision stability in this threshold interval.

The pharmacological treatment of PD frequently necessitates dopaminergic drug combination regimens, and dose escalation due to the fluctuating nature of symptoms during disease progression is susceptible to the triggering of cumulative effects. LEDD, as a standardized indicator of dopaminergic drug load, has been extensively utilized to evaluate clinical progression in PD (23). A meta-analysis demonstrated that factors such as increased LEDD and diminished quality of life were associated with PD-MCI (24). However, none of the studies that utilized MoCA to screen for risk factors for PD-MCI corroborated the independent predictive value of LEDD for the total MoCA score (25). This finding is consistent with the study referenced in this article, which failed to demonstrate significant predictive efficacy in a multifactorial model despite the possible correlation of LEDD with PD-MCI. This suggests that LEDD may be weakened due to clinical confounding factors such as movement complications or limitations in statistical power. The PDQ-39, a validated self-report scale, quantifies patients’ health-related quality of life through 39 entries, with higher total scores suggesting more severe quality of life impairment. Cross-sectional analyses revealed that the PDQ-39 total score was significantly higher in the PD-MCI group compared to the PD-NC group, suggesting that attention and language impairments are associated with a poorer quality of life (26). Consequently, in clinical practice, there is a greater imperative to adopt individualized drug delivery strategies, with a preferential selection of dopaminergic drugs that exhibit superior cognitive safety, and a regular evaluation of the dynamic relationship between the cumulative dose of drugs and cognitive function, in accordance with the guidelines established by the International Movement Disorders Association. Concurrently, life interventions should be implemented for patients with severely impaired motor function, in conjunction with physical therapy and the utilization of assistive devices to enhance the capacity to perform daily activities. It is to be posited that, in the final analysis, the cognitive decline may be delayed by a variety of intervention modalities.

In recent years, the “kidney-brain axis” theory, proposed by interdisciplinary research, has provided a new perspective on the pathogenesis of PD. Evidence suggests that renal α-syn oligomers can migrate through the circulation, cross the blood–brain barrier, and then induce the formation of Lewy bodies in the central nervous system through a prion-like transfer mechanism (27). A large prospective cohort study from Biobank UK confirmed that an impaired eGFR, estimated based on Cr and Cys C, is strongly associated with a significantly increased risk of developing PD (28). It has also been found in PD-MCI cohorts that decreased eGFR and decreased uric acid/creatinine ratio predict progressive cognitive decline in PD patients (29). These findings support the view that renal dysfunction is a key risk factor for PD and suggest that disruption of renal metabolic homeostasis may be an early driver of neurodegenerative disease. Cr is the traditional indicator of kidney function, reflecting muscle mass and renal excretion capacity, but it is susceptible to confounding by age, sex, diet and activity, and may mask the effects of early kidney disease on PD (30). In contrast, Cys C, which is produced stably and undisturbed by non-renal factors, is more sensitive to changes in GFR and is the biomarker of choice for assessing renal dysfunction (31). Indeed, studies have attempted to assess the relationship between renal function and PD using composite indicators such as uric acid/creatinine ratio (32) and microalbumin/creatinine ratio (33). However, while such composite metrics increase the sensitivity and dimensionality of the assessment to some extent, they may also obscure the direct relationship between a single renal function parameter and the pathomechanism of PD. There is only one large case–control study that included 3,797 patients with PD who were followed for 7 years. The results of the study showed that patients’ serum creatinine levels did not show a significant downward trend in the time window from 2 years before to 2 years after PD diagnosis, while only CRP levels showed some correlation with PD progression (34). This suggests that although combined indicators may reveal certain disease associations, the role of individual renal function indicators in the early detection and prediction of progression of PD needs to be clarified in further studies. Serum Cys C levels have been shown to be significantly elevated in patients with PD-MCI compared with those with PD-NC, and this indicator correlates with neurofilament light chain protein, a key biomarker of neuronal axonal damage (35). A cross-sectional study in a Chinese population further confirmed that elevated serum Cys C levels were not only associated with PD disease progression but also showed a more significant trend of elevation in the PD-MCI subgroup (14). It is worth noting that there is a lack of research evidence directly investigating the association between PD-MCI and Cr levels. Based on this, Cr and Cys C, two biomarkers that can accurately reflect renal function, were included in the prediction model construction in this study, and one-way regression analysis showed that both were potential risk factors for PD-MCI. However, after multifactorial correction, only Cr retained an independent predictive value, which may be related to the pathophysiological interactions mediated by the “kidney-brain axis” and is consistent with previous studies on the influence of renal metabolites on neurodegenerative processes.

Under the framework of the “kidney-brain axis,” the association between Cr and the risk of PD-MCI deserves in-depth study. When renal function is impaired, the neurotoxicity caused by the accumulation of uremic toxins may trigger PD-MCI. Moreover, impaired renal function may also interfere with the lymphatic drainage throughout the body and the central nervous system. Dysfunction of the lymphatic system will reduce the ability to clear metabolic waste in the brain, leading to the accumulation of harmful substances and exacerbating neural damage. DTI-ALPS is a new method proposed in recent years to assess glymphatic function in patients. Available research suggests a possible link between glymphatic dysfunction and CI in patients with PD (36). Studies have been conducted to differentiate between different levels of CI using the Summary Mental State Examination or MoCA scores, both of which confirmed that glymphatic dysfunction was significantly correlated with cognitive scores, suggesting that the function of the glymphatic system may influence cognitive levels (37, 38). Buccellato et al. (4) suggest that the glymphatic system is responsible for removing harmful substances such as α-syn, and that abnormalities in its function may directly contribute to the progression of PD. Current experiments in both PD patients and animals have shown significant changes in the glymphatic system of the brain, in particular reduced expression of aquaporin-4, a change that may affect the efficiency of waste removal in the brain. Imaging analysis also revealed that this association may involve multiple mechanisms of action in the inferior frontal gyrus, the orbital white matter structures of the anterior cingulate gyrus and the area of the blue patch (37, 39). Taken together, this evidence suggests that there are multiple lines of evidence supporting remodeling of the intracerebral glymphatic system in patients with PD-MCI. Chen et al. (40) refined the stratification of the PD group (NC/MCI/dementia) and found that the ALPS index was significantly lower in the MCI group compared with the NC group, and the index was negatively correlated with the degree of cognitive decline on a gradient, suggesting that glymphatic dysfunction may be associated with cognitive decline in a progressive manner. These lines of evidence suggest that the ALPS index not only serves as a novel imaging marker of PD-associated cognitive impairment but also influences cognitive trajectory by regulating the efficiency of metabolite clearance in the brain, which is both methodologically and mechanistically consistent with the core findings of the present study. Of course, there are conflicting findings in existing studies on the lateralization characteristics of the cerebral glymphatic system, with some studies finding an abnormal L-ALPS index in PD patients (41) and others examining the correlation between glymphatic function and motor symptoms suggesting a lower R-ALPS index than in the left hemisphere (42). Notably, the present study found a significant decrease in left hemisphere ALPS index in patients with right-handed PD-MCI, a spatial distribution characteristic that is consistent with the findings of a longitudinal study showing that baseline left-sided ALPS index could serve as an independent predictor of conversion of PD-MCI to dementia, as shown by the data of Pang et al. (43).

Although the L-ALPS index was not included in the multivariate model to avoid multicollinearity confounding in this study, univariate regression analysis showed that L-ALPS had a stronger predictive validity for PD-MCI than R-ALPS. This left hemisphere dominance effect may be related to the right-handedness of the enrolled patients, and its spatial distribution characteristics are anatomically consistent with the pattern of lateralization in PD neurodegenerative lesions (44).

It is well known that PD-MCI is an important transition stage in the progression from PD to PDD, and early identification of risk factors has a dual clinical value in slowing disease progression and improving patient prognosis. In this study, we constructed a multidimensional scoring model by integrating conventional clinical indicators and found that increased LEDD, increased PDQ-39 scores, increased Cr and Cys C levels, and decreased ALPS index were all risk factors for PD-MCI. Multifactorial logistic regression analysis confirmed that LEDD and Cr could be used as independent predictors of PD-MCI progression. The novelty of this study is that it is the first to combine the pathological mechanism of the kidney-brain axis with the theory of cerebral glymphatic system dysfunction, providing a new perspective on the cross-system interactions in the analysis of cognitive decline in PD. However, this study has several limitations. The proportion of excluded cases due to loss to follow-up or inability to cooperate with MR examination was relatively high (14.6%), which may lead to selection bias in the selection of included cases. Due to the single-center design and limited sample size, the statistical power of the correction for confounding may be affected. In addition, patients with cognitive impairment with MoCA scores below 18 were under-represented, resulting in reduced clinical applicability of the DCA curve at risk thresholds above 0.75. Future research should incorporate larger sample sizes derived from multicenter prospective cohorts. The application of machine learning algorithms could facilitate the integration of multimodal biomarkers and the development of dynamic prediction models, thereby improving the management of different cognitive impairment subgroups in PD. Additionally, prospective designs with optimized patient retention strategies, such as rapid MR sequences and sedation when necessary, should be implemented. The inclusion of sensitivity analyses would also be crucial to quantify and mitigate the impact of potential biases, ultimately enhancing the robustness and generalizability of the study findings.

In conclusion, we found that LEDD and Cr were independent predictors of PD-MCI. Meanwhile, we constructed an intuitive clinical column-line graph prediction model that integrated clinical features such as LEDD, PDQ-39 score, UPDRS I score, Cr, Cys C and ALPS index, blood biomarkers and neuroimaging indices to assess the risk of comorbid MCI in PD patients. The model showed good diagnostic efficacy in identifying PD-MCI.

## Data Availability

The datasets presented in this study can be found in online repositories. The names of the repository/repositories and accession number(s) can be found in the article/Supplementary material.

## Footnotes

^1^ https://trackvis.org/dtk/

^2^ https://fsl.fmrib.ox.ac.uk/fsl/fslwiki/FSLeyes
